# 外科医生和病理医生对胸腺恶性肿瘤切除标本的处理方法和程序

**DOI:** 10.3779/j.issn.1009-3419.2014.02.06

**Published:** 2014-02-20

**Authors:** Frank C. Detterbeck, Cesar Moran, James Huang, Saul Suster, Garre Walsh, Lawrence Kaiser, Mark Wick, 北京大学肿瘤医院 付浩

**Affiliations:** 1 Division of Toracic Surgery, Deportment of Surgery, Yale University School of Medicine, New Haven, Connecticut; 2 Department of Surgery, Toracic Service, University of Texas MD Anderson Cancer Center, Houston, Texas; 3 Department of Pathology, Memorial Sloan-Kettering Cancer Center, New York City, New York; 4 Department of Pathology, Medical College of Wisconsin, Milwaukee, Wisconsin; 5 Department of Toracic and Cardio-vascular Surgery, University of Texas MD Anderson Cancer Center, Hous-ton, Texas; 6 UT Health Science Center, Houston, Texas; 7 Department of Pathology, University of Virginia Health System, Charlottesville, Virginia

胸腺恶性肿瘤发病率低，临床病例分散，需要结合多中心经验才能进行良好的研究，但由于在诸多方面没有统一标准，使得研究与研究之间的可比性差。其中外科医生和病理医生对胸腺恶性肿瘤切除标本的处理是统一标准中至关重要的部分。针对目前胸腺瘤的常用手术方式胸骨劈开根治性R0切除术的标本处理作如下规定，包括切除胸腺瘤及周围的受累结构说明如何处理手术切除标本。活检或细胞学标本的处理另文描述^[1]^。本文首先由国际胸腺肿瘤协作组织（International Tymic Malignacy Interest Group, ITMIG）中病理医生和外科医生组成的小组回顾相关文献形成初步建议^[2, 3]^，交扩展组审议提炼后分发给ITMIG成员进讨论，再将ITMIG成员回馈的建议和几个大中心试点的情况进行提炼，最终形成的建议经ITMIG批准并被采用。因此，既强调证据又代表ITMIG成员的广泛共识。

## 1 术中标本处理的要求

### 1.1 问题与现状

目前对手术切除标本的通常的作法是，由外科医生切除肿瘤后，交由病理科处理并最终由病理科医生出报告了事。其实这样至少存在对离体肿瘤位置的定位和切缘表述的问题。首先，一旦肿瘤离体即使是外科医生也很难准确辨认标本的方向。其次，手术过程很容易破坏周围的结缔组织使其与原来的部位分离而发生位移。第三，对关注区域缺乏交流，如前所述一旦标本切除离体，病理科医生（事实上即使是手术医生）对哪些边缘是最需要关注的，和标本与术野的哪部分对应的关系就已经不能清楚的辨认与表述，例如在活体上很容易区别胸膜，但由于离体标本组织的退缩或者损伤，肉眼甚至是显微镜都很难辨认。最后，由于手术医生和病理医生缺乏面对面的交流，使组织破坏后关注区域没有得到足够的病理学检查与关注。表 1列出了手术医生与病理医生需关注与交流的问题。

**1:** 外科医生操作规范 Recommended routine policies for surgeons

标记
切除过程中标记关注区域，包括标本和患者
常规标记标本邻近心包和无名静脉的区域（若被联合切除，则标记这些结构）
常规标记标本左/右胸膜表面（若胸膜切除）
标记标本邻近上腔静脉的区域
缝线标记疏松结缔组织
方向
外科医生应参与标本方向定位
外科医生应与病理科医生使用一种通用的交流系统共同定位标本方向
推荐将切除标本按原来位置摆放在纵隔板或图纸上
推荐对切除标本拍数码照片
推荐对标本及其周围结构行素描并标记
淋巴结
胸腺瘤患者术中应清扫任何可疑转移淋巴结
对于Ⅰ期和Ⅱ期胸腺瘤，推荐行邻近和前纵隔淋巴结清扫
对于Ⅲ期淋巴结，推荐行系统性的前纵隔淋巴结清扫和对胸内某些部位系统性淋巴结采样，如气管旁、主肺动脉窗、隆突下等部位
对于胸腺癌，若前纵隔、胸腔内、锁骨上和下颈部淋巴结等部位淋巴结可疑转移或确定转移，至少应行系统性淋巴结采样
冰冻切片
对冰冻切片的解释需十分谨慎，而且仅适用于意外发现或怀疑为非胸腺肿瘤的患者，如淋巴瘤和生殖细胞肿瘤
利用冰冻切片判断切缘情况是十分困难的，其假阴性和假阳性率高，临床诊断应像镜下诊断那样得到重视
手术记录
手术记录应特别提到如下内容：
是否存在肿瘤肉眼残余，若有应记录部位
切除的范围，如完全胸腺切除
易分离粘连的表现和部位（不怀疑侵犯）
联合切除的结构（如纵隔胸膜、心包、膈神经和无名静脉）和器官（如肺）
术中关注的区域，包括如何标记标本和患者
探查淋巴结的区域和评估的程度，如采样或完全切除
是否胸膜和心包腔内有可疑转移
注：本表得到版权所有者© 2011 by the International Association for the Study of Lung Cancer复制许可。

### 1.2 建议

#### 1.2.1 定位

外科医生应边切除边对关注区域标记，以确保准确的辨认这些区域并进行病理检查。应在关注区域留置缝线标记，缝线不仅仅在组织表面，要带更深的组织结构（如果需要，甚至深到瘤体），这样可以防止在进一步的操作中破坏组织，反之如果缝线留置在疏松网状组织，那么缝线的位置可能改变。应该边切除边进行缝线标记，而不是在标本完全切除后标记。同时，手术野的相关区域也要用银夹标记，这样便于切除后辨认，以便于术后可能的放疗。理想的缝线标记线结应该宽松，以便于大体标本的切缘用染料标记后容易去除缝线。此外，建议某些特殊区域即使没有肉眼切缘阳性，也没有相应结构的切除，也要常规标记，对于较大的肿瘤标准的标记区域包括，邻近心包和无名静脉的标本表面，邻近上腔静脉和左右纵隔胸膜的表面。建议对每位患者都要在邻近无名静脉和心包的标本表面做标记。而对于小的肿瘤，对邻近上腔静脉或胸膜表面标记可能并不适用。在同一医疗中心用于标记的方法应该统一，例如用黑丝线标记常规区域，而用其他颜色标记特殊关注的区域。这样作的目的是突出特殊的关注的区域和能准确辨认有代表性的区域，便于常规的病理检查。然而，可能在标本完全切除后，由于结缔组织的破坏或退缩，在标本上又出现另外一些值得关注的区域，这些开始不被关注的区域也应受到特别的关注，且应在病理报告中注明“一个在术中未关注的区域”。这都需要病理科医生和外科医生在标本切除后送达病理科实验室之前及时交流。建议在切除时一旦发现这些破坏或变形的组织时就要留置缝线标记，防止进一步破坏。

#### 1.2.2 标本方向的确定

在手术室的时候就要开始合理的处理和评估切除的标本。由于外科医生是唯一了解标本与周围结构解剖关系的人，故外科医生要负责对标本方向正确定位、对受累结构标示、和对受累器官或关注区域进行说明。期望一个病理科技术员或实习生在接收到一个不规则标本后，尽全力重新构造标本与纵隔的空间关系，除了让我们得到更加困惑和错乱的结果，最终影响到治疗决策的制定外，将一无所获。

建议采用如同头颈外科常规应用的“颈板”标示头颈标本方向的方法一样，将标本摆放在“纵隔板”上用以确定标本的方向^[4]^。例如，在一个简单的软木板或蜡板上描绘出纵隔的轮廓（图 1），然后尽可能按照解剖关系小心将标本摆放在描绘的轮廓上。需要辨认与标本一起完整切除的受累结构，和指定临床关注的切缘（例如，使用缝线）。用这种方法将离体标本由手术室送到病理科，其方位仍然跟在人体内一样，而且每个潜在的受侵部位和临床相关的切缘很容易引起大家重视。建议将标本摆放在画有纵隔轮廓的纸板上进行数码照相（图 2）或素描（图 3），并保存在患者的病历资料里。

**1:**
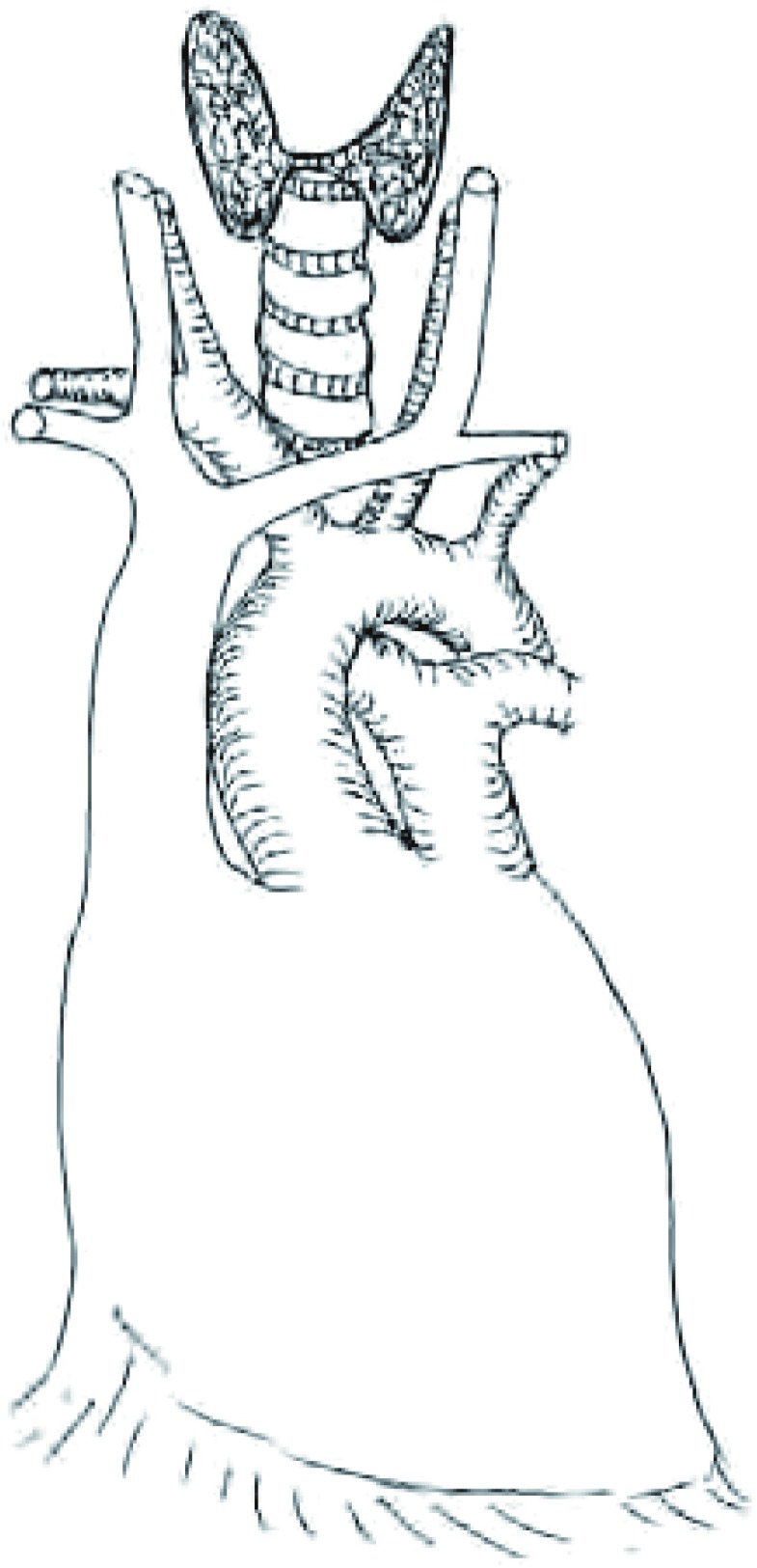
纵隔板，由软板和固定在上面的图纸组成，图像绘制比例与人体结构相似，用于保持切除标本的方向 Mediastinal board. A diagram on a soft board is useful in maintaining proper dimensions and orientation of specimens. Printing this figure as a full page corresponds roughly to the normal mediastinal dimensions and can be placed directly on a standard soft specimen board that is generally available in surgical pathology departments

**2:**
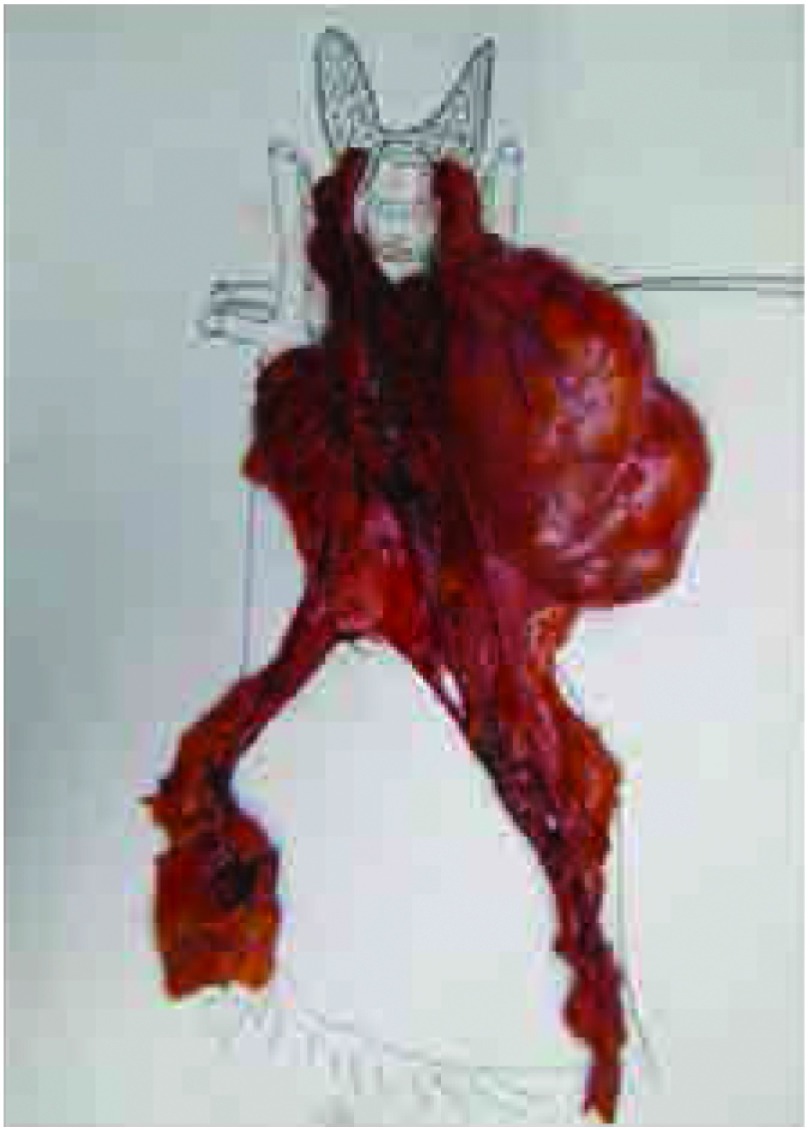
按体内解剖关系将标本摆放在纵隔板上，以体现肿瘤方向、与周围结构的关系和肿瘤的大小 Resected specimen oriented on the mediastinal board. Placement of the specimen and the paper diagram or board provides unambiguous orientation, depicts the rela-tionship to other mediastinal structures, and maintains the normal size of the resected tissue. Pins placed through the tissue into the board or paper diagram can be used to hold it in place if needed

**3:**
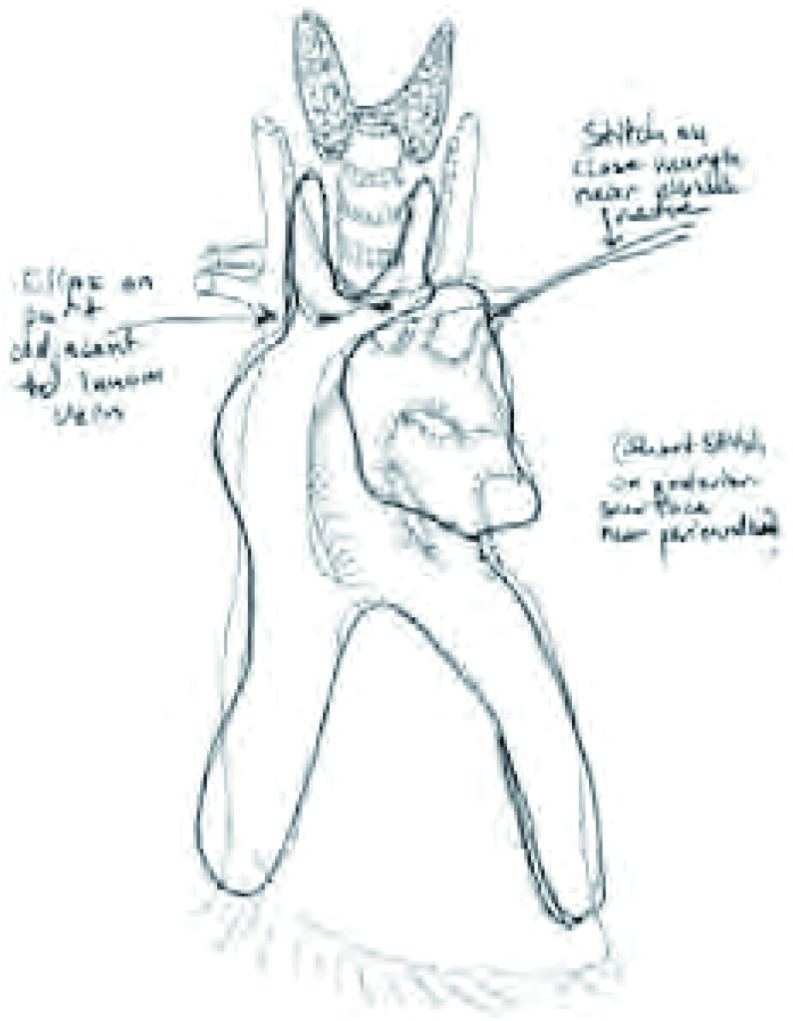
纵隔标本的素描。素描上的图解说明了标记区域和联合切除的结构，这有助于交流和减少困惑 Sketch of the oriented specimen on a mediasti-nal diagram. The sketch depicts marking stitches and addi-tional resected structures diagrammatically, which minimizes subsequent confusion and aids communication

标本的方位摆放应该由外科医生在手术室完成而不是在病理科由病理医生单独进行。有时，当标本离体后手术还在继续外科医生腾不出时间标记定位时，或种种原因病理科医生收到一个没有标记和定位方向的标本时，都需要及时和外科医生进行交流，并和外科医生或其指派参与手术的主要助手一起在手术室对标本进行标记和辨认方向。将标本展开摆放在纵隔板的图纸上，并附有标本的素描，这些方法可以增强交流。建议要么使用一个类似“纵隔板”方向定位系统，要么直接与外科医生面对面交流来辨认标本方位。缝线和银夹应该标记粘连的结构，如无名静脉、肺、或膈神经。更广泛的表面可以颜料标记，如双侧的纵隔胸膜或标本的前表面。外科医生在手术记录中必须一一列出切除的结构，而且每个关注的区域都应该在附带的病理检查要求中逐一记录。

#### 1.2.3 淋巴结采样或切除的要求

胸腺上皮肿瘤的类型不同淋巴结转移的发生率也不同，日本学者对来自115个中心1, 327例患者的研究发现，胸腺瘤淋巴结转移率为2%，其中Ⅰ期为0.4%、Ⅲ期为6%^[5]^，但该研究没有说明检查的淋巴结数量，以及是否进行淋巴结清扫或清扫哪些淋巴结；这项研究的多因素分析没有发现淋巴结状态是胸腺瘤的预后因素，而分期和R0切除是独立的预后因素；大约90%的淋巴结转移位于前纵隔，25%位于胸腔内，其中包括多个部位淋巴结转移的一些患者。由于各文献有关胸腺瘤淋巴结转移的研究程度不尽相同，得出的结果也颇为混乱，其中淋巴结缺乏预后意义的结论可能是源于样本量的不足。但是，淋巴结转移在胸腺癌和胸腺类癌中更常见，分别为27%和28%，发生在前纵隔的淋巴结转移二者分别为70%和90%，发生在胸腔其他部位的淋巴结转移分别为35%和60%，胸腔外的淋巴结转移均为30%。多因素分析表明，淋巴结转移状态和完全手术切除是胸腺癌和胸腺类癌的具有统计学意义的预后因素^[5]^。

要求在切除胸腺恶性肿瘤时，任何可疑的淋巴结如增大、或质硬、或PET/CT提示可疑者都应该清除、单独标记并送检。对于包膜完整的胸腺瘤建议清扫前纵隔淋巴结。对于侵犯邻近器官的Ⅲ期或Ⅳa期潜在可根治切除的胸腺瘤，建议常规清扫前纵隔淋巴结，并依据肿瘤位置的不同对胸腔内其他相应部位的淋巴结进行采样，包括气管旁、主肺动脉窗、隆突下等等。这主要基于如此采样的相对简单性，相当的转移发生率和获取准确淋巴结转移信息的必要性。对于胸腺癌，建议系统清扫，包括前纵隔、胸腔内、锁骨上和下颈部淋巴结。

## 2 冰冻切片的应用

冰冻切片诊断胸腺瘤相当困难，外科医生应该合理的使用而病理科医生也要谨慎报告，即使在富有经验的大中心冰冻切片诊断胸腺瘤也非常困难，对于那些经验有限的医疗机构更是如此。重视临床相关信息和有经验外科医生的反馈非常有帮助于做出正确诊断。外科医生必须明确意识到送检冰冻切片的纵隔肿瘤系活检小标本，常常受到人工挤压产生假象或不能代表整个病灶，因此活检时应利用各种手段加以避免。冰冻切片对诊断富含淋巴细胞的胸腺瘤更为困难，镜下不可能鉴别淋巴母细胞淋巴瘤和淋巴细胞为主胸腺瘤，尽管临床表现上前者进展快，而后者呈惰性表现。梭形细胞胸腺瘤是另一经常与其他发生在纵隔的梭形细胞肿瘤混淆的类型，例如孤立性纤维瘤、滑膜肉瘤或其他肿瘤。从冰冻切片上鉴别不典型胸腺瘤，即世界卫生组织（World Health Organization, WHO）分类的B3型胸腺瘤，和高分化鳞状细胞胸腺癌也非常困难，外科医生也需要知道病理科医生不能从形态上鉴别原发性胸腺癌和来自其他部位的转移癌^[6]^。囊性肿瘤在冰冻切片上评估尤为困难，因为胸腺组织纤维化和炎症改变经常掩盖恶性成分，需要等待石蜡切片加以鉴别。

庆幸的是，大多数情况下胸腺瘤手术中不需要冰冻切片明确诊断，因为临床上常能于术前通过诸如细针穿刺（fine-needle aspiration, FNA）细胞学检查和带芯穿刺检查明确诊断。术中冰冻切片可用于在其他手术（如冠脉搭桥术）中偶然发现的胸腺瘤或对其他纵隔肿瘤的评估。或对不可切除的纵隔肿瘤送冰冻切片以判断是否有足够的诊断组织。当外科医生在术中发现肿瘤不具备典型的胸腺瘤特征时，如，比预计的侵犯更严重、或区域坏死、或肿瘤的部位不典型等情况时，冰冻切片可有一定的诊断价值。当对侵袭性肿瘤进行根治性切除时，冰冻切片可以在一定程度上帮助外科医生判断切缘是否足够。但外科医生需要知道，实际上病理科医生也很难准确报告出这些内容，因为尽管还没有准确的数据，但胸腺瘤冰冻切片有着较高的假阳性和假阴性率，冰冻切片的结果不能替代经验丰富的外科医生的临床判断。

## 3 大体标本的准备与取材

在固定处理标本前应该根据不同的颜色的染料标记辨明标本的前后左右（图 4A、图 4B）^[3]^。这应该在标本切除的过程中或在手术室、或在大体病理实验室完成。在进行病理处理之前，外科医生和病理医生之间要直接交流解决所有没有弄清楚的问题。当肿瘤的不同解剖部位和标记明确后，建议从前向后像切面包片一样将肿瘤切成薄片（图 5）；建议以环周方式切开肿瘤展现整个肿瘤的环周。每块切片要按顺序摆放并用字母或数字标记，为石蜡包埋和进一步组织检查做准备。确定了标本的不同部位，如不同的染料颜色，和切片的顺序后很容易准确判断各切缘的情况。未受累的标本部分也要随机作常规切片检查（图 6）。

**4:**
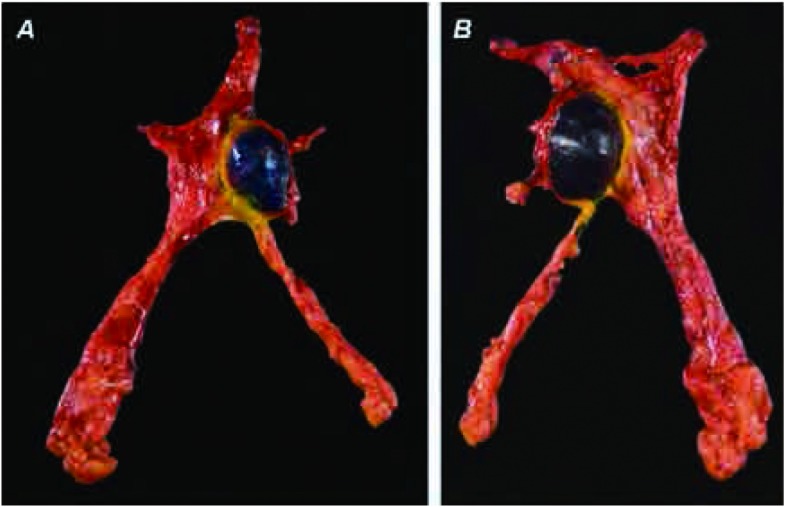
A：染色标本前面观。蓝色标记肿瘤前面部分、黄色标记右边部分。B：染色标本后面观。黑色标记肿瘤后面部分，黄色标记区域对应A图标记区域 A, Inked specimen, anterior aspect. Blue ink labels the anterior aspect and yellow ink the right lateral aspect of the tumor (on the left side of the photo-graph). B, Inked specimen, posterior aspect. The posterior aspect of the specimen has been inked with black ink. Note the yellow ink labeling the right lateral aspect of the tumor on the right side of the figure

**5:**
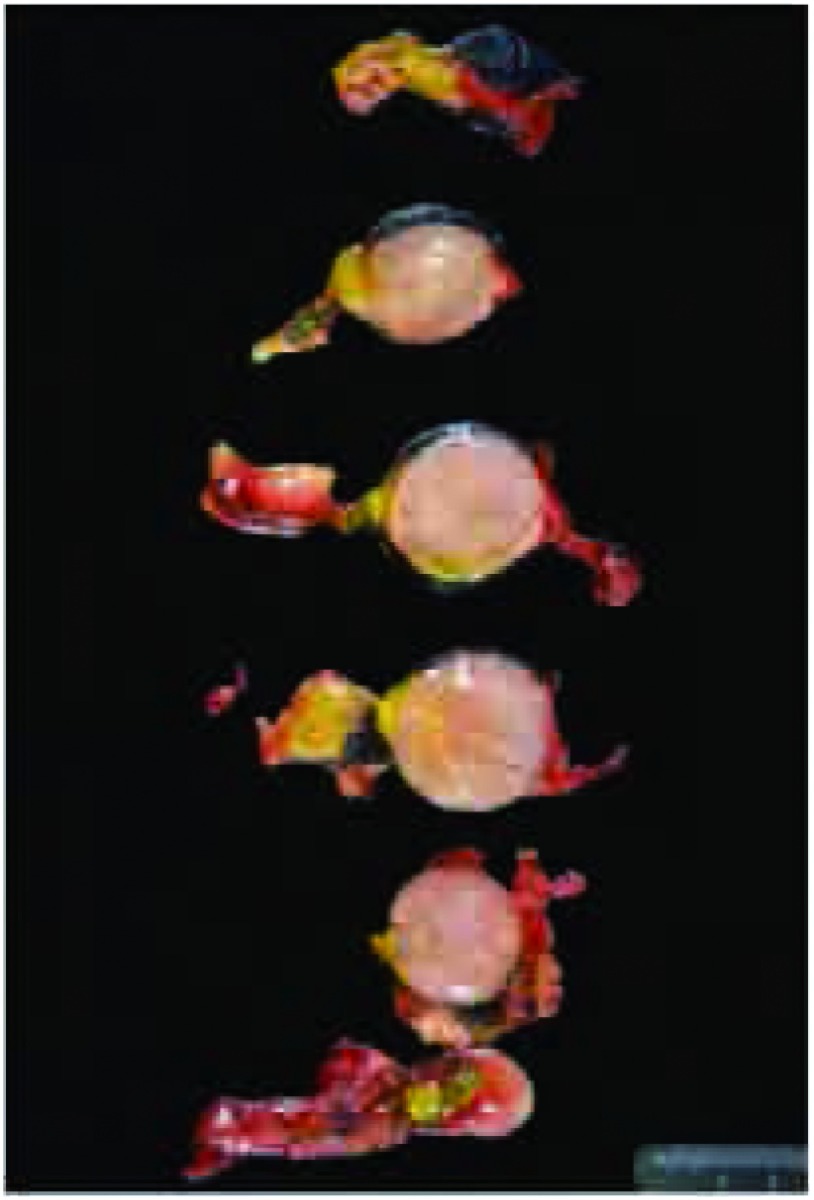
面包片样切开标本。从前至后将标本面包片样切成薄片，有助于记录肉眼特征和辨认需要特别关注和需要病理评估的区域。从图中可以看到，染料标记的区域很容易辨认 Bread-loafed tumor specimen. The tumor mass has been sectioned from superior to inferior in a bread-loaf manner into thin sections. This is useful to document specific gross features and to identify specific areas that may equire special attention and histopathological assessment. Note that the different colors are easily identifiable

**6:**
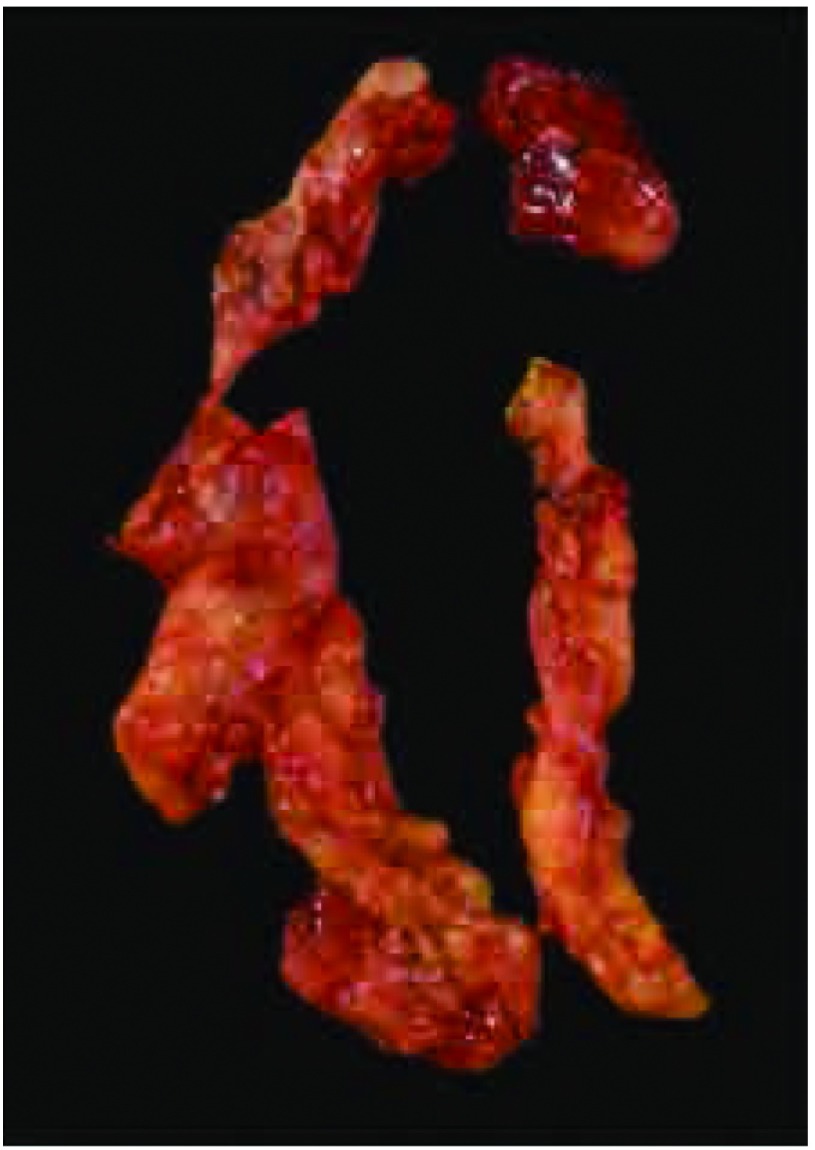
保留未受累组织，对其随机切片作病理评估 Remaining uninvolved thymic tissue. Random sections of this tissue should be obtained for histopathological assessment

镜下潜在不均一性是胸腺瘤的特点，同一个肿瘤的不同部位可有不同的组织学表现^[7]^。常规方法是在肿瘤标本的长径上每厘米至少取一块组织，如10 cm的肿瘤就需要取10块，5 cm的肿瘤则需要5块等等^[2, 3]^。这种方法在评估其他异质性肿瘤中已经有成功的经验，例如生殖细胞肿瘤和软组织肉瘤^[8-11]^。此外，过去的研究表明至少5块组织的系统采样能提高胸腺上皮肿瘤病理诊断的可靠性^[7, 12]^，因此美国外科病理解剖主任协会建议至少取5块组织^[3]^。如果胸腺肿瘤非常小，可以将其分成2块或3块整体镜检。对于大于5 cm的肿瘤，至少切成5块^[7]^。

储存组织以备将来研究对提高我们对这些肿瘤的生物学特征的认识至关重要。组织储存应由负责该病例的病理医生完成，他可以权衡在满足诊断的基础上尽可能多的为研究保留组织。由于胸腺瘤手术标本通常体积都较大，对于大多数病例来说保存组织简单可行。多数较大标本可以取1立方厘米的组织贴标签后速冻、或低温冰箱存储以备将来不同的用途。将组织行3 mm厚的连续切片福尔马林浸泡可用于常规组织学检查和石蜡包埋。肿瘤特殊部位的选择取决于病理医生的仔细肉眼评估和外科医生提供的相关信息。对标本所有表面都要常规查看，当然对外科医生或病理科医生怀疑切缘受累的地方一定要取材。此外，任何大体表现与其他部位明显不同的地方均应该取材。有完全不同的诊断的肿瘤在一个标本中共存的情况，如胸腺癌与胸腺瘤共存^[13]^。胸腺瘤的一个特殊亚组是广泛的甚至完全的囊性变^[14-16]^，这可能由于肿瘤供血不足而自发产生，也可能由诸如术前化疗等医源性原因导致^[17, 18]^。在没有术前活检的情况下病理科医生必须在囊肿周围广泛取材以获取用于诊断的组织，这对排除其他起源于胸腺解剖位置的囊性肿瘤很有必要，如生殖细胞肿瘤、或转移癌、或少见的恶性淋巴瘤等^[16]^。

## 4 镜下报告

### 4.1 侵犯

每个标本的最终病理诊断一定要包括肿瘤是否有侵犯，建议使用局限、微浸润或浸润等术语，见表 2。侵犯但没有侵透包膜不能定义为肿瘤侵袭，这类肿瘤也应归为局限性胸腺瘤。肿瘤必须侵透包膜才能定义为侵袭，浸润脂肪的深度小于3 mm且没有破坏标本涂染的外表面和侵犯周围组织结构者应定义为微浸润胸腺瘤。尽管区分肿瘤非浸润和微浸润的临床意义还不明确，但是这三层定义与当前的权威部门的建议是一致^[3, 19]^。此外，以往文献对非浸润和微浸润的定义模糊，对这些文献综合比较很困难，因此需要使用统一和明确的定义来进行前瞻性研究。记录肿瘤是否存在纤维包膜也很重要，必须认识到包膜是肿瘤引起的机体反应性的纤维粘连，而并不是天生存在的解剖标志。在一些病例中纤维包膜要么完全缺如要么部分包绕肿瘤，对肿瘤纤维包膜缺如的情况要在报告中明确记录为“胸腺瘤，部分未覆盖包膜”，和注明“肿瘤包膜侵犯不能在包膜缺如的区域评估”。对于肿瘤在没有包膜的地方与周围脂肪组织接触的情况要谨慎解释。病理科医生应该记录肿瘤包膜缺如的区域是否达到了标本染色的边缘，这不影响侵袭性的分类，但是定义为切缘阳性。

**2:** 病理科医生操作常规 Recommended routine policies for pathologists

大体标本准备
病理医生与外科医生需在术中交流，以解决存在的问题
切片之前需辨认关注区域
辨认处理过程中受破坏的区域
应明确辨认标本的前、后、左、右部分，如标记的不同颜色
按从前往后的顺序连续面包片样切开标本并送检
每隔1 cm切片都应送检
无论肿瘤直径大小，至少送检5块有代表性区域组织
对于未受累的胸腺组织应随机切片并送检
在满足病理诊断的情况下，应尽可能多的保留组织。
切缘报告
包膜完整和侵犯
胸腺瘤，局限性（包膜完整，尽管部分包膜可能缺失）
胸腺瘤，微浸润（侵透包膜，但侵犯邻近脂肪组织深度 < 3mm)
胸腺瘤，侵袭性（浸润包括纵隔脂肪在内的周围结构）
切缘情况
切缘阴性
肿瘤被完全正常的组织覆盖
侵犯结构但有空腔分界，如胸膜或心包
肿瘤累及标本染色表面，但包膜完整
肿瘤在有组织破坏的区域暴露，但该区域不是术中关注的区域，这种情况需单独记录
切缘阳性
肿瘤累及染料标记的手术切缘
与最近切缘的距离
是否 < 3 mm
如果 < 1 mm或 < 1 hpf, 至少应再检查三个切面
新辅助治疗后标本的处理及报告
大体标本的准备与初始切除标本一致
无论肿瘤直径大小，至少送检5块有代表性区域组织
每隔1 cm切片都应送检
完全病理缓解的诊断需要按初始切除标本的处理方法仔细采样
应根据对多张标本切片评估后的结果，报告残余肿瘤的百分比（以10%递增的方式）
注：本图得到版权所有者© 2011 by the International Association for the Study of Lung Cancer复制许可。

鉴别肿瘤与胸膜或心包是致密的粘连、或是部分侵犯、或是穿透这些结构是非常困难的，建议尽可能对这些情况进行鉴别^[19]^。建议对于这些粘连但没有侵犯的情况，根据病理科医生的最佳判断，归类为“肿瘤粘连但未侵犯”纵隔胸膜或心包。

### 4.2 切缘状态

切缘状态包括分清切除组织的切缘与相应周围组织、或与体腔表面相应结构界面的关系，如纵隔胸膜、或心包、或无名静脉、或上腔静脉腔内的内皮等。肿瘤达到纵隔胸膜或心包但周围仍有空腔界限时不应定义为切缘阳性；肿瘤不但延伸至相应体腔而且侵犯到邻近的其他器官，但切除标本显示肿瘤没有侵犯到染色边缘时也定义为切缘阴性，反之则应定义为切缘阳性。

胸腺肿瘤的一个问题是肿瘤周围几乎经常没有组织，有的话也是退缩的疏松结缔组织而且很容易被人为破坏。外科技术分离的结缔组织构成了手术切缘，错误报告切缘的一个常见原因是对这些周围组织少的肿瘤区域随意下结论。任何有可能关注的切除区域应该由外科医生按照如前所述的方法特别标记和命名。标本外层表面染色如果发现在包膜上，或是出现在一层薄的结缔组织上而没有跟肿瘤接触即应定义为切缘阴性。很多情况下胸腺瘤的包膜构成了标本的外表面。对于这些病例肿瘤穿透包膜达到染色的外表面就应该定义为切缘阳性。

对于所有≤3 mm的较近的切缘需要记录肿瘤与染色边缘的最近距离。此外，当切缘≤1 mm建议至少在该区域获取3个层面的切片并在报告上记录，因为很可能多几个层面的检查会发现切缘阳性。通过不同颜色染料标记，结合标本方向和术中对关注区域缝线标记等方法可以明确报告切缘或近切缘状况，对于阳性切缘应该注明来自标本的哪部分。外科医生标记的任何区域都要特别的检查和报告。

关于术中没有发现但是记录有组织破坏区域的报告，要特别注意。应根据前面提到的方法，描述这些区域。然而，如果发现暴露的肿瘤不是术中关注的而是发生了组织破坏的区域，应该划分为阴性，并进一步说明，如阴性，非关注区域，而是组织破坏暴露肿瘤。

### 4.3 新辅助治疗后结果报告

术前诱导治疗后切除的大体标本制备和直接手术切除的原则是一样的，切缘状态的报告也是一样。但是，新辅助治疗后肿瘤有可能发生坏死。因此，新辅助治疗后的大体标本病理检查可能需要更多的切片，以保证能有效代表组织学表现。仍然鼓励建立组织库，但是由于一些原因，建立组织库可能比较困难，对许多研究可能不适用。肿瘤的特征可能会受新辅助治疗的影响而改变，除非有诱导治疗前的样本作比较，不然对病理结果解释是非常困难的。

在新辅助治疗引起肿瘤囊性变的病例中，应该报道残余肿瘤的比例。残余肿瘤的比例是通过对连续排列的大体标本切片肉眼检查后估计的一个比例，并记录在大体描述中。肿瘤的残余部分必须整体取材用于组织学检查，并且将标本画成图片和拍成数码照片。这些区域应该与组织学部分相结合，以便于更进一步评估残余肿瘤组织的比例。术前放疗或化疗将造成肿瘤的退缩和消失，被纤维结缔组织所取代^[20]^。在这些病例中，只有组织学检查才能评估残余肿瘤的范围。不建议使用本文以外的方法评估是否达到完全病理缓解。

## 5 讨论

本文推荐的胸腺瘤外科切除标本的病理报道方法，代表了ITMIG的共识。这些方法可以防止在发表的文章中出现模糊定义，增强不同研究之间的可比性。这些方法以目前可获得的数据为基础，如果这些方法不适用，可采用胸腺肿瘤或其他肿瘤用过的方法。在没有可利用方法的情况下，也可采用单纯根据逻辑法推演的方法。

这些推荐的方法将被ITMIG正式采用，而且将被作为国际肺癌研究会（International Association for the Study of Lung Cancer, IASLC）胸腺肿瘤分期的基础。各医疗中心是否采用这些方法的关键在于，能否利用它们准确判断切缘状况，并判断切缘阳性部位在患者体内对应的位置（以辅助化疗），和这样做是否能改善复发率。

采用这些规范的方法可以促进个人研究，而且有助于探索其它外科和病理处理方法。应该认识到，这些方法仅代表发展更科学可靠方法解决胸腺恶性肿瘤问题过程中的一个步骤。希望随着时间的推移这些方法能得到改进或被更好的方法所替代。

## 6 结论

在外科医生和病理医生处理胸腺瘤切除标本上，本文推荐的方法代表了一个广泛共识。其中一些方法是以之前的研究为基础的，而另外一些则基于合理的逻辑推理，并未经研究证实。这些方法的广泛采用，促进了不同的数据的整合，增强了可比性，有利于进一步研究。在公认有效的方法提出之前，全面收集数据是很有必要的，不仅包括胸腺肿瘤的病理报告，还应包括其分期和治疗信息。

## References

[1] Marchevsky A, Marx A, Strobel P (2011). Policies and reporting guide-lines for small biopsy specimens of mediastinal masses. J Torac Oncol.

[2] Marchevsky AM, Hammond EH, Moran C (2003). Protocol for the examination of specimens from patients with thymic epithelial tumors located in any area of the mediastinum. Arch Pathol Lab Med.

[3] Weydert J, De Young B, Leslie K (2009). Recommendations for the reporting of surgically resected thymic epithelial tumors. Am J Clin Pathol.

[4] Cole I, Hughes L (1997). The relationship of cervical lymph node metastases to primary sites of carcinoma of the upper aerodigestive tract: a patholog-ical study. Aust NZ J Surg.

[5] Kondo K, Monden Y (2003). Lymphogenous and hematogenous metastasis of thymic epithelial tumors. Ann Torac Surg.

[6] Moran CA, Suster S (2008). Thymic carcinoma: current concepts and histologic features. Hematol Oncol Clin North Am.

[7] Moran CA, Suster S (2000). On the histologic heterogeneity of thymic epithelial neoplasms. Impact of sampling in subtyping and classification of thy-momas. Am J Clin Pathol.

[8] Steeper T, Mukai K (1984). Solid ovarian teratoma: an immunocytochemical study of 13 cases with clinicopathologic correlation. Pathol Annu.

[9] Niezabitowski A (2001). Current problems in clinicomorphological assessment of soft tissue tumors. Pol J Pathol.

[10] Levin H (1993). Prognostic features of primary and metastatic testis germ-cell tumors. Urol Clin North Am.

[11] Kenney RJ, Cheney R, Stull MA (2009). Soft tissue sarcomas: current management and future directions. Surg Clin North Am.

[12] Hasserjian R, Strobel P, Marx A (2005). Pathology of thymic tumors. Semin Torac Cardiovasc Surg.

[13] Suster S, Moran CA (1996). Primary thymic epithelial neoplasms showing combined features of thymoma and thymic carcinoma: a clinicopath-ologic study of 22 cases. Am J Surg Pathol.

[14] Moran C, Suster S (2001). Thymoma with prominent cystic and hemorrhagic changes and areas of necrosis and infarction; a clinicopathologic study of 25 cases. Am J Surg Pathol.

[15] Suster S, Moran C (1995). Malignant thymic neoplasms that may mimic benign conditions. Semin Diagn Pathol.

[16] den Bakker M, Oosterhuis JW (2009). Tumours and tumour-like conditions of the thymus other than thymoma; a practical approach. Histopathology.

[17] Hejna M, Haberl I, Raderer M (1999). Nonsurgical management of malignant thymoma. Cancer.

[18] Kurup A, Loehrer SP (2004). Thymoma and thymic carcinoma: therapeutic approaches. Clin Lung Cancer.

[19] Detterbeck FC, Nicholson AG, Kondo K (2011). The Masaoka-Koga stage classification for thymic malignancies: clarification and definition of terms. J Torac Oncol.

[20] Macchiarini P, Chella A, Ducci F (1991). Neoadjuvant chemotherapy, surgery and postoperative radiation therapy for invasive thymoma. Cancer.

